# Telemonitoring for Chronic Heart Failure: Narrative Review of the 20-Year Journey From Concept to Standard Care in Germany

**DOI:** 10.2196/63391

**Published:** 2024-12-04

**Authors:** Sebastian Spethmann, Gerhard Hindricks, Kerstin Koehler, Stefan Stoerk, Christiane E Angermann, Michael Böhm, Birgit Assmus, Sebastian Winkler, Martin Möckel, Mirja Mittermaier, Monika Lelgemann, Daniel Reuter, Ralph Bosch, Alexander Albrecht, Stephan von Haehling, Thomas M Helms, Stefan Sack, Tarek Bekfani, Jan Wolfgang Gröschel, Magdalena Koehler, Christoph Melzer, Jan Wintrich, Bettina Zippel-Schultz, Georg Ertl, Claus Vogelmeier, Nikolaos Dagres, Jasmin Zernikow, Friedrich Koehler

**Affiliations:** 1 Department of Cardiology, Angiology and Intensive Care Medicine Deutsches Herzzentrum der Charité (DHZC) Berlin Germany; 2 Charité-Universitätsmedizin Berlin Corporate Member of Freie Universität Berlin and Humboldt-Universität zu Berlin Berlin Germany; 3 DZHK (German Centre for Cardiovascular Research), Partner Site Berlin Berlin Germany; 4 Department Clinical Research & Epidemiology, Comprehensive Heart Failure Centre, Department of Internal Medicine I University Hospital Würzburg Würzburg Germany; 5 Comprehensive Heart Failure Centre University Hospital Würzburg Würzburg Germany; 6 Department for Internal Medicine I University Hospital Würzburg Würzburg Germany; 7 Department of Internal Medicine II, Cardiology, Marien Hospital Herne, Ruhr University Bochum, Bochum, Germany. Herne Germany; 8 Department of Cardiology and Angiology University of Giessen Giessen Germany; 9 DZHK (German Centre for Cardiovascular Research), Partner Site Rhein/Main Kerckhoff Heart Center Bad Nauheim Germany; 10 Department of Internal Medicine and Cardiology BG Klinikum Unfallkrankenhaus Berlin Berlin Germany; 11 Department of Emergency and Acute Medicine Charité - Universitätsmedizin Berlin Berlin Germany; 12 Department of Infectious Diseases, Respiratory Medicine and Critical Care Charité-Universitätsmedizin Berlin Berlin Germany; 13 Gemeinsamer Bundesausschuss Berlin Germany; 14 Cardio Centrum Ludwigsburg-Bietigheim Ludwigsburg Germany; 15 Kardiologische Gemeinschaftspraxis Wilmersdorfer Strasse 62 Vivantes Klinikum Am Urban Berlin Germany; 16 Department of Cardiology and Pneumology University of Göttingen Medical Center Göttingen Germany; 17 German Center for Cardiovascular Research (DZHK), Partner Site Göttingen Göttingen Germany; 18 German Foundation for the Chronically Ill Berlin Germany; 19 Peri Cor Cardiology Working Group / associated UCSF Hamburg Germany; 20 Department of Cardiology, Pneumology, and Internal Intensive Care Medicine Schwabing Hospital, Academic Municipal Hospital Munich Munich Germany; 21 Department of Cardiology and Angiology Universitätsklinikum Magdeburg Magdeburg Germany; 22 ECRC Experimental and Clinical Research Center Charité – Universitätsmedizin Berlin, corporate member of Freie Universität Berlin and Humboldt-Universität zu Berlin Berlin Germany; 23 Working Group on Cardiovascular Magnetic Resonance Experimental and Clinical Research Center, a joint cooperation between Charité Medical Faculty and the Max-Delbrück Center for Molecular Medicine Berlin Germany; 24 Department of Preventive Sports Medicine and Sports Cardiology University Hospital ‘Klinikum rechts der Isar’, School of Medicine Technical University Munich Munich Germany; 25 Department of Acute Medicine Donauspital Wien Wien Germany; 26 Department of Internal Medicine, Pulmonary and Critical Care Medicine Member of the German Center for Lung Research (DZL) University of Marburg Marburg Germany

**Keywords:** telemedicine, e-counseling, heart decompensation, Europe, patient care management

## Abstract

**Background:**

Chronic heart failure (CHF) is a major cause of morbidity and mortality worldwide, placing a significant burden on health care systems. The concept of telemedicine for CHF was first introduced in the late 1990s, and since 2010, studies have demonstrated its potential to improve patient outcomes and reduce health care costs. Over the following decade, technological advancements and changes in health care policy led to the development of more sophisticated telemedicine solutions for CHF, including remote patient management through invasive or noninvasive telemonitoring devices, mobile apps, and virtual consultations. Years of public funding in Germany have generated evidence that remote patient management improves outcomes for patients with CHF, such as quality of life, and reduces hospital admissions. Based on these data, the Federal Joint Committee (Gemeinsamer Bundesausschuss; G-BA) decided, independently of the current European Society of Cardiology recommendations, to incorporate telemedicine as a standard digital intervention for high-risk patients with reduced left ventricular ejection fraction in Germany in 2020.

**Objective:**

This review aims to illustrate the journey from the initial concept through pioneering studies that led to telemedicine’s integration into standard care, and to share current experiences that have positioned Germany as a leader in cardiovascular telemedicine.

**Methods:**

We review and discuss existing literature and evidence on the development and implementation of telemonitoring for CHF in Germany over the past 20 years. Relevant studies, reports, and guidelines were identified through a comprehensive search of electronic databases, including PubMed, Google Scholar, and specialized journals focused on CHF telemonitoring.

**Results:**

Pioneering studies, such as the TIM-HF2 (Telemedical Interventional Management in Heart Failure II) and IN-TIME (Influence of Home Monitoring on Mortality and Morbidity in Heart Failure Patients with Impaired Left Ventricular Function) trials, demonstrated the effectiveness of remote patient management applications for patients with CHF in Germany and their applicability to current practices involving both invasive and noninvasive methods. Collaborations between researchers and technology developers overcame barriers, leading to sustainable improvements in patient care. Ongoing research on artificial intelligence applications for prioritizing and interpreting individual health data will continue to transform digital health care.

**Conclusions:**

The establishment of telemedical care for patients with HF across Europe is likely to benefit from experiences in Germany, where significant improvements have been achieved in the care of patients with HF.

## Introduction

### The Origins of Telemedicine

The history of telemedicine is closely tied to the technical innovations of its era, beginning in the 19th century with Samuel Morse’s invention of the telegraph and Morse code system. The introduction of the telephone by Philipp Reis in 1861, and its subsequent development by Alexander Graham Bell in 1876, opened new possibilities for telemedicine, enabling remote diagnosis for the first time. In 1911, radio technology was first used in maritime shipping for telemedical consultations. Since 1931, Cuxhaven Hospital in Germany has provided medical advice to seafarers via radio [1].

Over the past 150 years, advances in telecommunications have driven new telemedical applications. However, it is primarily in the last 20 years that telemedicine has been increasingly used for the outpatient care of chronically ill patients. Since the advent of smartphones in 2007, telemedicine has become part of daily life, supporting both asynchronous care—through electronic exchange of telemedical data—and real-time consultations via video or telephone conferencing.

Telemedicine thus has 2 primary application scenarios: on the one hand, it facilitates professional exchanges between geographically distant health care providers (known as “doc-to-doc telecardiology”). On the other hand, it enables a direct connection between doctors and patients within the patient’s home environment, using information and communication tools (referred to as “doc-to-patient telecardiology”).

In Germany, the implementation of telemonitoring was notably delayed due to restrictive legislation, including a long-standing legal ban on remote treatment, which was only eased in 2018. Although direct doctor-patient contact remains the standard practice, telemedical cocare is now an available option. However, a complete replacement of outpatient care with telemedicine remains excluded.

All other fundamental requirements—such as the personal delivery of services, adherence to specialist standards, medical duty of care, patient confidentiality, and the obligation to inform patients of treatment risks—must also be upheld in telecardiological cotreatment and are outlined in a treatment contract. A particular requirement is that patients, most of whom lack medical training, must be able to perform simple diagnostic procedures (eg, measuring blood pressure and recording electrocardiograms [ECGs]) with adequate precision.

### Telemedicine in Heart Failure: A Life Prolonging and Economic Digital Measure

Cardiac decompensation is the most common and prognostically significant complication of chronic heart failure (CHF), with an average survival time of about 2.5 years following its first occurrence [2]. Each day, approximately 1250 patients are admitted to hospitals in Germany due to cardiac decompensation [3]. Since 2005, it has been the leading single diagnosis for hospital admissions, representing a substantial economic burden on the health care system [4].

The decline in cardiac function often begins gradually and can progress to life-threatening decompensation, marked by changes in specific vital parameters. With modern sensor technology, early shifts in these vital signs can be detected remotely and transmitted immediately to the attending physician, enabling an effective doctor-to-patient telemedicine care model. The first evidence of the efficacy of home telemonitoring as a preventive strategy was provided by the 2005 TEN-HMS study (Trans-European Network-Home-Care Management System). The study demonstrated a reduction in the average hospital stay by 6 days and a significantly lower 1-year mortality rate (29%) compared with usual care (45%) in patients with CHF and a left ventricular ejection fraction (LVEF) below 40% [5].

**Figure 1:**
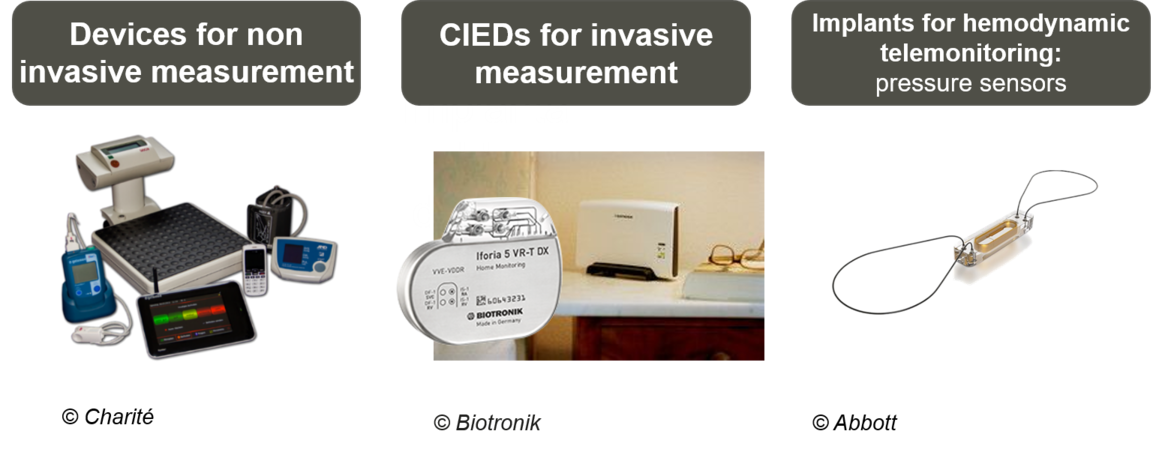
Selected types of sensors used in telemonitoring. CIED: cardiac implantable electronic device.

Telemonitoring can be categorized as either invasive or noninvasive, based on the type of sensors used to record vital signs (Figure 1). In invasive telemonitoring, disease-specific vital signs are monitored using active or passive implanted devices. In noninvasive telemonitoring, patients record vital signs daily using external home measuring devices, such as scales, blood pressure monitors, external ECG devices, or wearables. Through active self-measurement, patients provide treating physicians with up-to-date information on their current health status.

**Figure 2:**
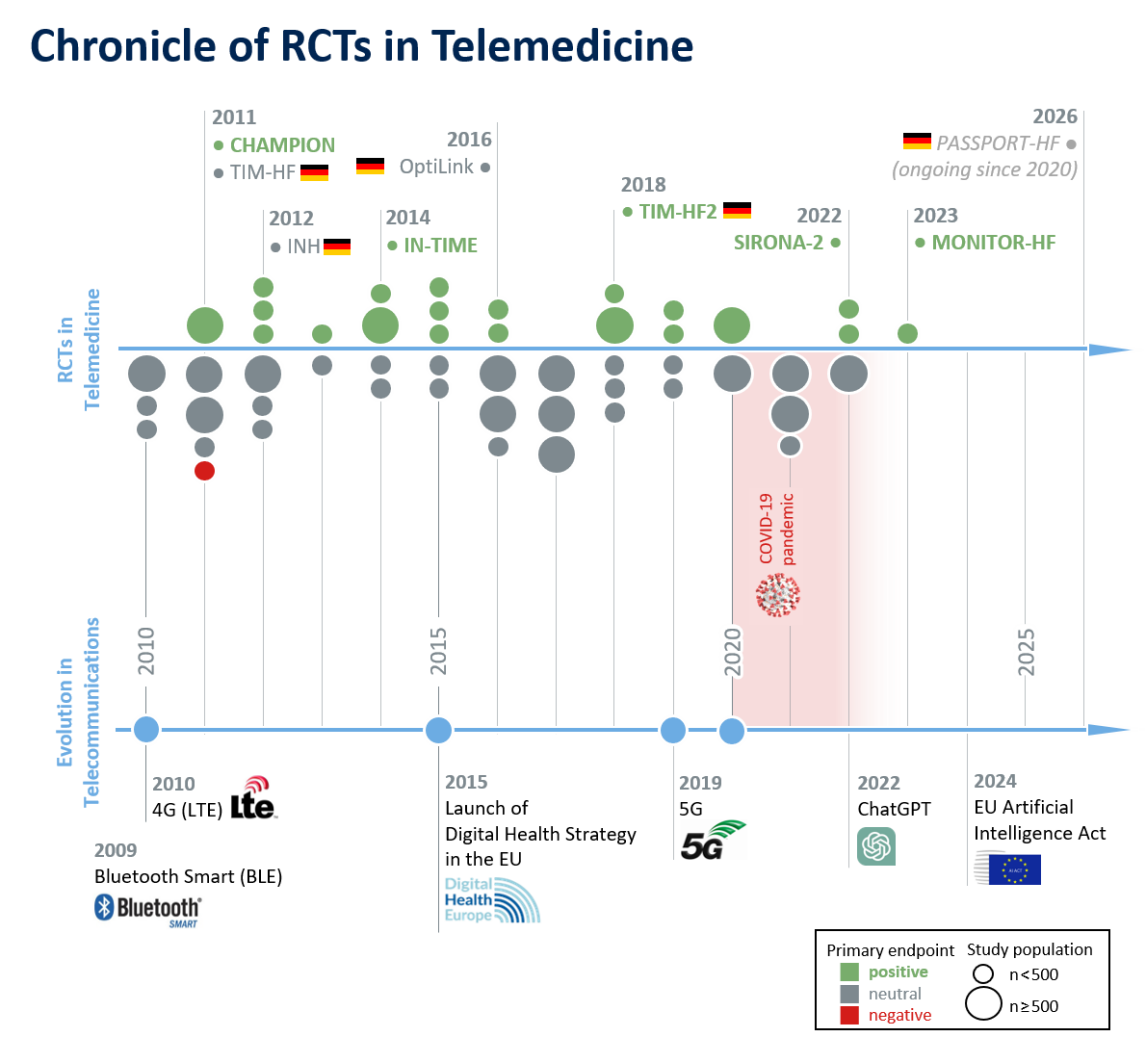
Highlighted trials and randomized controlled trials (RCTs) on telemonitoring from 2009 to present.

Among the 6 landmark studies (Figure 2) on the efficacy of cardiovascular telemedicine, 3 were conducted in Germany, 2 of which received public funding. These studies ultimately verified telemedicine as an independent treatment method. As a direct result, the German Federal Joint Committee (Gemeinsamer Bundesausschuss; G-BA) has included telemedicine as a digital intervention for high-risk patients with heart failure (HF) with reduced left ventricular ejection fraction in the benefits catalog in Germany.

This overview presents the German perspective, tracing the journey from the initial study idea through evidence generation to implementation in standard care. It reports on current experiences in Germany and offers insights into future developments.

## Methods

This narrative review synthesizes existing literature and evidence regarding the development and implementation of telemonitoring for CHF in Germany over the past 20 years. Relevant studies, reports, and guidelines were identified through a comprehensive search of electronic databases, including PubMed, Google Scholar, and specific journals focused on telemonitoring for CHF.

The selection of literature was guided by its relevance to the topic, emphasizing major studies, clinical trials, and policy papers that significantly contributed to the evolution of telemedicine in HF care. Sources were not restricted to randomized controlled trials; observational studies, expert opinions, and case studies were also included to provide a comprehensive understanding of the field.

The review process involved critically analyzing and summarizing the findings, focusing on key milestones, technological advancements, and their impact on clinical practice. Additionally, insights from ongoing and future research initiatives were incorporated to highlight emerging trends and potential future directions in telemedicine for HF care.

## Results

### Evidence on the Efficacy of Noninvasive Telemonitoring

#### Overview

Noninvasive devices, such as scales, blood pressure monitors, ECG devices, and wearables, have demonstrated benefits in multiparametric data transmission, ease of use, integrative monitoring of comorbidities (eg, diabetes), and cost-effectiveness.

Evidence for the efficacy of noninvasive cardiovascular telemonitoring first emerged in the mid-2000s, leading to a differentiation between trials based on invasive or noninvasive sensor technology. Three major landmark trials—INH (Interdisciplinary Network for Heart Failure), TIM-HF (Telemedical Interventional Monitoring in Heart Failure Study), and TIM-HF2 (Telemedical Interventional Management in Heart Failure II)—were conducted in Germany and received public funding from the German Ministry of Education and Research, as well as the Federal Ministry for Economic Affairs and Climate Protection.

#### The INH-Study Program (2004-2022)

The randomized, controlled, multicenter INH (Interdisciplinary Network for Heart Failure) study program (ISRCTN23325295) investigated the effects of regular telephone-based monitoring and education provided by HF nurses as part of an intensified care approach by a specialized HF center. The remote patient management strategy emphasized patient self-monitoring of vital parameters and HF symptoms. The first phase of the trial assessed the 6-month risk of mortality and morbidity in 715 patients with an LVEF of ≤40%, all of whom had been hospitalized for acute decompensated HF.

The composite primary outcome for time to all-cause death or rehospitalization was neutral between the telemonitoring group (n=352) and the usual care control group (n=363). This observation was influenced by a significant reduction in all-cause mortality to 38% in the remote patient management group, which was associated with a trending increase in hospitalization rates in the intervention arm. Additionally, patients in the telemonitoring group demonstrated improvements in quality of life, functional class, and adherence to medical therapy.

A long-term evaluation of the INH trial (the “E-INH study”) in a larger population (n=1022) demonstrated that telephone-based monitoring over an 18-month period led to a significant reduction in all-cause and cardiovascular mortality after 120 months in the group of survivors who had received remote patient management (33% vs 40%; *P*=.04). Additionally, HF-related and cardiovascular hospitalizations were significantly reduced at 18, 36, and 60 months by –25%, –29%, and –30%, respectively. As previously noted, the telemonitoring procedures did not include automated transmission of patient-recorded vital signs. Instead, they relied on postdischarge telephone monitoring, where specialized nurses reviewed patients’ self-monitored results. These nurses also provided information, education, and guidance on optimal medical therapies; advised on appropriate actions in case of suspected HF decompensation; supported self-adjustment of diuretic agents; and coordinated specialist care as needed.

Overall, the INH study program suggests that telephone-based monitoring and education alone can improve short-term quality of life and mortality, with a sustained long-term impact demonstrated by reduced all-cause and cardiovascular mortality, fewer hospitalizations, and improved quality of life [6,7].

#### The TIM-HF Study Program (2005-2024)

The TIM-HF study (NCT00543881) and the TIM-HF2 study (NCT01878630) were large, randomized, controlled, open, parallel telemedical trials that investigated the efficacy of noninvasive, multiparameter telemonitoring in a population with moderate to severe HF (New York Heart Association [NYHA] class II or III).

Both studies were fully publicly funded. The core element of this telemedical care model was a telemedical center staffed with experienced HF specialists and nurses, offering regular and emergency telemedical services 24 hours a day, 7 days a week, functioning as a “virtual emergency department” [8].

The TIM-HF trial was a telemedical randomized controlled trial conducted between 2008 and 2010 across 4 German federal states: Berlin, Brandenburg, Saxony-Anhalt, and Baden-Württemberg. The main inclusion criteria were an LVEF below 35% and a history of HF hospitalization within 24 months before randomization. The primary endpoint was all-cause mortality. The study concluded on a predetermined date (April 30, 2010), resulting in varying follow-up periods for each patient, with a minimum of 12 months and a maximum of 28 months (mean follow-up 21.5 months, SD 7.2 months). The results for the primary endpoint were neutral [9]. However, a post hoc analysis identified a subset of patients who may benefit most from telemonitoring [10].

TIM-HF2 was a nationwide telemedical randomized controlled trial conducted between 2013 and 2018 across 13 German federal states. The main inclusion criteria were informed by the post hoc analysis of the TIM-HF study. Patients were enrolled regardless of their LVEF status if they had experienced an HF-related hospitalization within 12 months before randomization. A total of 1538 patients were randomized equally into either a telemedical intervention arm or a control arm. The study follow-up period was 1 year. The primary endpoint was defined as “days lost due to unplanned cardiovascular hospitalization or death within 1 year.” The secondary endpoint was total mortality. Telemedical intervention showed a significant benefit in the primary endpoint, with 17.8 days lost in the telemedical group compared with 24.2 days in the control group (*P*=.046). Additionally, there was a nearly 30% reduction in 1-year mortality in the telemedical group, with a mortality rate of 7.9% per 100 patient-years (95% CI 6.14-10.10) compared with 11.3% per 100 patient-years in the control group (95% CI 9.21-13.95). This corresponded to a hazard ratio of 0.70 (95% CI 0.50-0.96; *P*=.028) [11].

The 2 subsequent TIM-HF studies offered robust evidence supporting the efficacy of noninvasive telemonitoring in a specific subgroup of patients with HF.

### Evidence on Invasive Telemonitoring Using Cardiac Implantable Electronic Devices

#### Overview

Active implants, also known as cardiac implantable electronic devices (CIEDs), not only serve their primary function of treating cardiac arrhythmias through defibrillators or cardiac resynchronization therapy, but also provide an option for telemonitoring. In the context of telemonitoring, these devices periodically transmit data on physical activity, submit ECG readings, and may detect changes in pulmonary impedance. However, this active home monitoring application is limited to a specific subgroup of patients with HF who meet the eligibility criteria for CIED therapy.

The IN-TIME (Influence of Home Monitoring on Mortality and Morbidity in Heart Failure Patients with Impaired Left Ventricular Function) landmark trial marked the beginning of an era characterized by technology-based, industry-funded study designs, moving away from reliance on governmental funding. This shift has paved the way for the generation of fundamental evidence and has further shaped the research landscape in telemedicine. As a result, companies providing device therapy have become involved as principal investigating parties in telemedical research.

#### The IN-TIME Trial (2007-2014)

The IN-TIME trial (NCT00538356) is currently the only study that has investigated device-based multiparameter telemonitoring using active CIEDs and has reported a positive primary endpoint for a composite score that includes all-cause mortality.

This international multicenter randomized trial was conducted at 36 centers across Israel (2 sites), Australia (1 site), and Europe (33 sites), including 26 sites in Germany, and was funded by BIOTRONIK SE & Co. KG. A total of 664 patients with congestive heart failure classified as NYHA class II-III, with an LVEF of less than 35%, who had undergone implantation of a dual-chamber CIED, either an implantable cardiac defibrillator (ICD) or a cardiac resynchronization therapy with a defibrillator (CRT-D), were randomized in a 1:1 ratio to either the standard care control arm or the device-based telemonitoring in addition to standard care arm.

The telemonitoring software installed in the CIEDs transmitted daily tracked data to the manufacturer’s online platform. These data included not only arrhythmic events but also parameters related to the device’s functionality. For the intervention group, any subsequent abnormalities were further forwarded to the study centers, where HF nurses conducted structured interviews to assess typical HF symptoms and discussed potential interventions with physicians.

With the primary endpoint defined as a “modified Packer score,” which includes the incidence rate of all-cause mortality, HF-related hospitalizations, changes in NYHA class, and quality of life assessments, the intervention group receiving telemonitoring demonstrated a significantly lower occurrence rate of the composite clinical endpoint compared with the control arm (18.9% vs 27.2%, respectively; *P*=.013). Importantly, total mortality was significantly lower in the telemonitoring arm compared with the control arm (3.0% vs 8.2%; hazard ratio 0.36; *P*=.004). However, the rates of hospital admissions for worsening HF did not differ significantly between the 2 groups. In a post hoc analysis, no interactions were found between the effect of telemonitoring and device type (ICD or CRT-D), NYHA class at enrollment, or age (≤67 or >67 years), indicating a consistently significant impact of telemonitoring across all subgroups.

Thus, the IN-TIME trial demonstrated the beneficial impact of device-based telemonitoring in patients with HF through daily data transmission. Consequently, these results may not be directly transferable to remote monitoring platforms that utilize less frequent data transmissions [12].

#### The OptiLink-HF Trial (2009-2015)

In the invasive telemonitoring OptiLink-HF study (Optimization of Heart Failure Management using Medtronic OptiVol Fluid Status Monitoring and CareLink Network; NCT00769457), a total of 1002 patients were enrolled following implantation of an ICD or CRT-ICD [13]. These CIEDs also included the capability to measure intrathoracic impedance. In the event of a significant decrease in thoracic impedance, indicative of pulmonary fluid overload, the devices transmitted fluid threshold crossing alerts.

Patient enrollment occurred across 65 centers in Germany from October 2008 to April 2013. The mandatory inclusion criteria for participants included CHF classified as NYHA class II or III, an LVEF of less than 35%, and at least one of the following: an HF hospitalization within the past 12 months, the use of intravenous diuretic medication for outpatient treatment, or an increase in natriuretic peptide levels within the preceding 30 days.

Patients were randomized in a 1:1 ratio, with the telemedicine function either turned on or off. Physicians were required to respond within 2 working days using a prespecified intervention algorithm [14]. During these phone contacts, discussions included changes in body weight, medication adjustments, and symptoms of decongestion. The initiation of therapeutic measures was at the discretion of the physicians, allowing for a range of responses from no action to reprogramming the fluid threshold crossing alert threshold. If fluid threshold crossing alerts continued for more than 12 days, patients were prompted to attend an in-office or in-hospital visit for further evaluation.

The follow-up period for all study participants was set at 18 months, with an option for reinformed consent to extend follow-up until the last patient completed their study visit. This led to a mean follow-up duration of 22.9 months for the participants.

The primary endpoint of the study was the rate of death from any cause and the rate of cardiovascular hospitalizations. Notably, the primary composite endpoint and its individual components were not achieved. Major limitations of the study included technical issues, with 24% of the impedance alarms failing to transmit to the treating physicians. Additionally, inappropriate responses to the alerts were observed in 40% of cases, which included instances of no patient contact at all (12.4%); patient contact exceeding the 2-working-day threshold (10.8%), particularly during weekends and public holidays (4.0%); and a lack of appropriate medical intervention (32.8%).

Interestingly, a post hoc analysis of the OptiLink-HF trial suggested that impedance-based telemonitoring could have significant prognostic effects when alert transmissions were managed appropriately [15]. Additionally, the intervention was found to significantly decrease the primary endpoint in patients with preserved renal function, while it did not demonstrate the same benefit in those with chronic kidney disease [16]. This highlights the critical importance of timely and effective management of alert transmissions, especially in these vulnerable patients [17].

### Trial-Based Evolution of Invasive Hemodynamic Telemonitoring

#### Overview

The alterations in intracardiac filling pressures, which serve as the ideal surrogate for incipient congestion, are not well reflected by changes in body weight. Invasive hemodynamic telemonitoring provides the advantage of earlier detection of impending cardiac decompensation events [18]. However, regardless of the underlying technical devices used, reliable and prompt integration into the workflows of telemedical centers is considered crucial for achieving therapeutic effectiveness.

Intracardiac filling pressures can be monitored by tracking the pulmonary artery pressure (PAP), right ventricle, or left atrium. These implants enable the transmission of tracked values in a “passive” manner upon query with a wireless external readout device.

#### The CardioMEMS HF System

The electrode- and battery-free PAP sensor CardioMEMS (Abbott Laboratories) is implanted in a proximal branch of the pulmonary artery and calibrated to the individual patient. Changes in pulsatile flow are converted into a pressure derivative and wirelessly transmitted to a remote dashboard, such as that at a telemedical center. Daily measurements must be initiated actively by the patient, and specialized staff regularly interrogate the PAP trends accumulating over time to determine whether and which actions need to be taken. In the Abbott-funded CHAMPION trial (NCT00531661) [19], conducted in the United States and Canada, 550 patients in NYHA functional class III, regardless of LVEF, had PAP sensors implanted. The CHAMPION trial reported a 39% risk reduction in HF-related hospitalizations after 6 months in patients who received physician-led interventions based on the assessed PAP values compared with the control group [19]. These effects were sustained after 12 months and remained consistent in the nonrandomized 3-year extended follow-up period [20]. The prospective MEMS-HF registry (CardioMEMS European Monitoring Study for Heart Failure) had an observational design and yielded comparable effects when utilized within the European health care system [21].

These findings were corroborated by the pre–COVID-19 impact analysis of the GUIDE-HF trial (NCT03387813), which studied 1000 patients in NYHA class II or III from the United States [22], and the recent MONITOR-HF trial (NTR7673), conducted in 348 NYHA class III patients in the Netherlands [23]. Both trials indicated a sizeable benefit of PAP-guided management on HF hospitalization rates compared with the control group.

#### The CardioMEMS HF System in Germany: The PASSPORT-HF Trial

As aforementioned, the G-BA evaluates medical innovations for inclusion in the medical services covered by health insurance. Given the major differences in health care systems between the United States and Germany, the G-BA made a novel decision to directly fund a telemedicine randomized controlled trial across 50 study centers. The still ongoing PASSPORT-HF trial (Pulmonary Artery Sensor System Pressure Monitoring to Improve Heart Failure Outcomes; NCT04398654) aims to recruit 554 patients in NYHA class III who have experienced an HF hospitalization within the last 12 months.

PASSPORT-HF is investigating whether PAP-guided care, supported by HF nurses and utilizing daily monitoring of PAP values with the CardioMEMS system (Abbott), reduces the number of unplanned HF-related hospitalizations or all-cause mortality after 12 months.

In the PASSPORT-HF trial, patients with CHF are being randomized in a 1:1 ratio to either device implantation or, in contrast to the CHAMPION trial, no device implantation in the control arm. Both groups will receive structured telephone support from HF nurses in addition to guideline-compliant pharmacotherapy, which exceeds standard practice in Germany. Therefore, PASSPORT-HF aims to evaluate whether the previously reported benefits of PAP-guided hemodynamic monitoring as a novel HF management tool in routine outpatient telemedical care can be replicated in the German health care setting. Initial study results are expected in 2026 [24].

#### Recent Innovations: The CordellaHF Management System

The *Cordella HF Management System* (Cordella, Endotronix Inc.) comprises an implantable, battery-free PAP sensor and an external handheld patient reader. This system was evaluated in the SIRONA study program (NCT03375710).

The Cordella HF Management System is the first to facilitate the simultaneous recording of both invasive PAP data and externally measured parameters, such as blood pressure, heart rate, body weight, and oxygen saturation. This capability offers a more comprehensive overview of a patient’s compensation status to the HF care team. Additionally, the system enables patients to access their trending data through an easy-to-use handheld device for PAP measurement. Unlike the CardioMEMS HF system, the Cordella HF Management System allows for PAP measurements to be taken in various body positions and under stress conditions. This flexibility is particularly important, as patients have expressed a clear preference for measurements conducted while seated [25].

The *Cordella system* was initially evaluated in a first-in-human study involving 15 patients, where the PAP difference measured 90 days after implantation showed no significant difference between the values obtained from the Cordella sensor and those from right heart catheterization. Notably, patient compliance with daily remote monitoring exceeded 98% [26]. Following this, the SIRONA-2 multicenter, single-arm, open-label study (NCT03375710) included 70 patients with HF in NYHA class III across 4 sites in Ireland and Belgium, as well as 3 centers in Germany.

The primary efficacy endpoint that compared the *Cordella* PAP sensor measurements with established catheter-derived reference pressure at 90 days was met (mean PAP difference 0.0-2.9 mmHg, *P*=.003), remaining in good agreement for up to 12 months of observation. There were no reports of sensor failures or deaths within the 90-day period [25], and improvements in NYHA class and 6-minute walk distance were observed [27]. Hence, the *Cordella* management system appears feasible and safe and is currently under investigation in the PROACTIVE-HF single-arm study (NCT04089059), which has prespecified safety and effectiveness endpoints to provide objective evidence of a similar risk-benefit profile to the *CardioMEMS* system in 450 patients in NYHA class III (Figure 2).

### The Regulatory Process: From Trials to Standard Health Care in Germany

Telemonitoring in patients with CHF, as a digitally supported type of care, has been recognized as an independent examination and treatment method by the G-BA in March 2021 [28]. Consequently, telemonitoring became a standard health care service for this patient group in Germany [29].

Since 2009, health insurance has been compulsory for all German residents, as ruled by the German Insurance Contract Act [30]. As of 2022, approximately 87% of residents are covered by statutory health insurance, while about 10% have private insurance [31]. Both types offer telemonitoring as a service; however, private insurers define new aspects regarding current indication criteria (Textbox 1).

**The current inclusion criteria for heart failure telemonitoring (according to Gemeinsamer Bundesausschuss decision and reimbursement for private insurance).**

- New York Heart Association II-III stage heart failure with a left ventricular ejection fraction <40%.
- Implanted cardiac device (implantable cardiac defibrillator or cardiac resynchronization therapy with a pacemaker/defibrillator) or heart failure hospitalization due to cardiac decompensation in the past year.
- Present treatment according to current guidelines (guideline-directed medical therapy).
- Absence of identifiable factors that compromise the transfer of monitoring data or that would interfere with patient self-management.
- If other prerequisites are fulfilled: patients with private health insurance who have chronic heart failure and exhibit a left ventricular ejection fraction >40%; also including hospitalization for decompensated heart failure within the last 12 months.

Reimbursement by statutory health insurance companies requires a prior assessment of benefit, necessity, and cost-effectiveness. For the evaluation of digital care services such as telemonitoring, the G-BA has the statutory task of conducting a qualitative benefit assessment. The submission of research projects for assessment by the G-BA is subject to a particularly strict “1-shot” strategy, meaning that projects can only be submitted once.

Research on the efficacy of telehealth interventions in the care of patients with CHF is inconsistent. Reasons for the differing clinical endpoints are variations in the telemedical systems used (invasive measurement sensors vs noninvasive monitoring) and different concepts of guided care, such as the involvement of HF nurses. Additionally, the response times to abnormally transmitted values varied, with some studies defining responses during regular consultation hours while others provided 24/7 care through a telemedicine center.

Despite these differences in study design, the German TIM-HF2 landmark trial, among others, identified high-risk patients with a 12-month history of HF-related hospitalization as the population that benefits most from telemedical cocare.

The fact that there is no treatment benefit in the absence of such a recent HF hospitalization is an important evidence-based selection criterion for the indication of telemonitoring, thereby avoiding unnecessary resource use [29].

Since 2016, the Institute for Quality and Efficiency in Health Care (Institut für Qualität und Wirtschaftlichkeit im Gesundheitswesen [IQWiG]), as the highest joint self-governing body of the German health care system, has been investigating the benefits of invasive telemonitoring with active implants and noninvasive telemonitoring in CHF on behalf of the G-BA. The aforementioned IN-TIME trial for invasive telemonitoring and the German TIM-HF and TIM-HF2 trials for noninvasive telemonitoring formed the basis of an assessment regarding additional health care benefits [11,12,32]. In its assessment of October 2019, the IQWiG identified a statistically significant reduction in cardiovascular mortality through telemonitoring as a sufficient additional benefit [33].

According to the aforementioned G-BA decision, telemonitoring is currently only indicated for patients with CHF with reduced LVEF. This selection criterion is inconsistent with current trial data, which also revealed a benefit for patients with CHF with preserved LVEF [11]. A reevaluation by the G-BA and, ultimately, by the IQWiG for translation into medical care is currently pending.

### The Role of Medical Scientific Societies in Telemonitoring Implementation Processes

The German Cardiac Society (Deutsche Gesellschaft für Kardiologie [DGK]) provides an important basis for the development of collaborative projects in telemedicine for patients with CHF. In 2005, the DGK founded a telemedicine working group (Working Group 33) that also addresses the certification criteria for telemedicine centers [34]. Accordingly, a position paper on the certification processes ensuring quality standards for telemedical centers, aligned with legal requirements, was published in 2022 [35].

A thorough understanding of the available technical capabilities is essential for the effective collaboration of different study groups and the development of joint projects. Therefore, the working group on CHF (Working Group 10) developed guidelines for patients planning long-distance travel [36]. As a straightforward and intersecting approach to telemedical surveillance, remote monitoring of implanted devices (such as pacemakers, ICDs, and implantable cardiac monitors) has been recommended and further implemented by the European Society of Cardiology (ESC) [37-39].

Another important step toward representing professional interests at the national level was the establishment of the “Digital Transformation in Internal Medicine” Commission of the German Society of Internal Medicine (Deutsche Gesellschaft für Innere Medizin [DGIM]) in 2019. The DGIM, founded in 1882, is the largest medical society in Germany, steeped in tradition with over 30,000 members [40-42]. First, in 2014, the DGIM published guiding principles for the implementation of telemedical service provision [43]. The commission further serves its self-conception as a platform to impart literacy, offer training opportunities, and provide scientific support services.

From a European perspective, the ESC defined telemedical care concepts in a position statement in 2015 as 1 of 7 application areas of eHealth in cardiology [44]. As a result of technical developments and updated evidence, this paper was considered preliminary; therefore, the *ESC Digital Health Committee* was established in 2016 and is continuously discussing scientific updates in this area. The recent guidelines of the ESC on the diagnosis of acute HF and CHF [45] differentiate between hemodynamic, invasive telemonitoring using implants that measure PAP and noninvasive telemonitoring with external measuring devices. This distinction has led to the issuance of class II-B recommendations for both telemedical technologies.

### International Cooperations

The German research and development project focused on telemedicine for patients with HF has always been linked to international advancements in this area. A notable example is the German-Austrian project “Telemed5000” (project number FKZ-01MD19014A), which explored the applicability of artificial intelligence (AI) within the workflow of a telemedical center. With a budget of approximately €2.4 million (US $2.6 million), this project was funded by the German Federal Ministry of Economic Affairs and Climate Action and cofunded by the Austrian Federal Ministry of Climate Action, Environment, Energy, Mobility, Innovation, and Technology [46].

There has been and continues to be an intensive international exchange regarding scientific results related to telemedicine in patients with HF, as well as insights from Germany’s implementation process, particularly with neighboring European Union countries. For instance, the *German Foundation for the Chronically Ill* (Deutsche Stiftung für chronisch Kranke) organized a roundtable of experts from 7 European countries—France, Germany, Italy, Poland, Spain, the Netherlands, and the United Kingdom—in December 2023. This gathering aimed to review the current state of telemedicine in the participating countries, facilitate mutual learning, and provide impetus for European cooperation.

Establishing reliable regulations, overcoming regional differences, redefining roles and processes, personalizing health care services, promoting innovation and research, utilizing AI, and efficiently managing and safeguarding health care data have been identified as key levers for the further development of telemedicine.

### Current Practice in Germany

An estimated 150,000-200,000 patients with CHF in Germany are currently eligible for telemedical cocare [47]. Additionally, the G-BA in Germany has defined criteria for the inclusion of current cardiovascular telemonitoring (Textbox 1).

Accordingly, care is provided by the primary physician (either a primary care doctor or a specialist in internal medicine or cardiology) in cooperation with a consulting physician (cardiologist) at the telemedicine center. Therefore, the entire care process is designed for outpatient settings only and is completely separated from in-hospital stakeholders.

Cardiologists who wish to provide telemonitoring must hold a license for treating patients within the framework of public health insurance. Additionally, the establishment of a telemonitoring center requires the presence of a technical infrastructure and a license for the follow-up of CIEDs [28,48].

**Figure 3:**
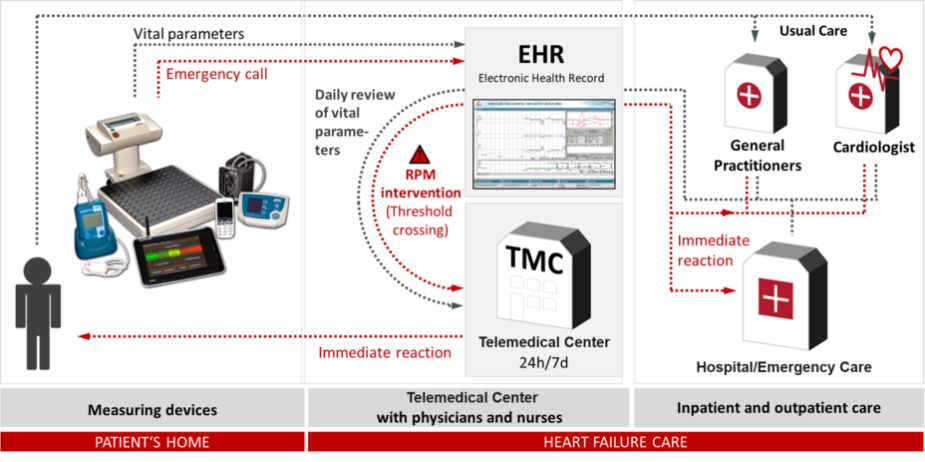
Components of telemonitoring in heart failure according to the G-BA (Federal Joint Committee/Gemeinsamer Bundesausschuss) approval in Germany.

After a patient has consented to telemonitoring, qualified HF nurses conduct training on the proper use of the issued equipment. This training is essential for ensuring adequate patient adherence, which forms the foundation for accurate measurements and subsequent data transfer. By contrast, the monitoring of CIEDs is automated, with individually programmed alerts transmitted as soon as a connection is established between the CIED and the transmitter. An 80% data transmission rate is mandatory and must be reported by the telemedicine center annually. The center is required to evaluate patient data at least 5 days a week or daily for severely ill patients under “intensified monitoring.” If a response is necessary, such as a change in medication, the telemedicine center is obligated to contact the primary care physician or interact directly with the patient if the physician is unavailable. These responses must adhere to specified time frames: the processing of an alert must not exceed 24 hours, while the transmission of a subsequent clinical need for action should reach the primary attending physician within a maximum of 48 hours (Figure 3).

Furthermore, a quality assurance program for telemonitoring has been implemented, focusing on adherence to guidelines, daily data review, alarm management, and tracking hospitalizations for decompensated HF and other relevant scenarios.

### Further Digital Applications in Germany

Digital health applications offer prescription-enabled mobile apps that differ from classic telecardiology by providing daily evaluations of transferred values and deriving subsequent actions. These apps constitute the third pillar of patient management, incorporating mobile health (mHealth), and primarily aim to empower patients to manage chronic diseases through lifestyle and behavioral modifications.

The central website of the German Federal Institute for Drugs and Medical Devices (Bundesinstitut für Arzneimittel und Medizinprodukte [BfArM]) offers a directory of available digital health apps [49] categorized into 12 different medical fields, providing relevant information for both patients and health care providers. To date, only 1 Digital Health Application (DiGA), “ProHerz,” has been provisionally approved for patients with HF. However, it is recognized that long-term engagement with digital apps is achieved by only a small number of users; for instance, the 30-day retention rates for patients using mental health apps were only 3.3% [50].

To enhance adherence, future “DiGAs 2.0” that incorporate AI applications promise feasible transmission of vital parameters tracked by wearable devices. This advancement could facilitate more effective therapies in the remote analysis of HF compensation but necessitates thorough scientific evaluation. The initial narrative of a DiGA designed for sole use by patients is currently being reevaluated to explore higher scalability for remote monitoring and a more detailed integration into the medical practices of general practitioners and specialists.

### Outlook on Upcoming Challenges

#### Unmet Questions of Remote Patient Monitoring

Telemonitoring is an effective technology regarding availability, accessibility, and affordability. However, improvements in outcomes for patients with CHF through telemedicine have not been universally demonstrated across all available technologies and devices. Refining the current evidence remains challenging due to unresolved questions about the most appropriate monitoring technology for individual patients, the optimal time to initiate telemonitoring, and, most importantly, the duration of monitoring following cardiac decompensation [51].

Furthermore, there is a need to incorporate current evidence for special patient populations, such as patients with HF with preserved ejection fraction [52], into the catalog of services provided by statutory health insurance without requiring a complete restart of the authorization process.

In contrast to the introduction of a new drug therapy, transferring a digital therapy from a controlled study setup to routine health care is significantly more complex. To facilitate the introduction of noninvasive telemedicine in HF, the publicly funded prospective phase IV registry study, “TIM-HF4” (Telemedical Interventional Management in Heart Failure 4), will commence in 2025. This study aims to investigate patient acceptance and the effectiveness of noninvasive telemonitoring in HF under real-life conditions, focusing on outcomes such as repeated hospitalizations due to HF, death from any cause, and health economic criteria.

The transferability of telemonitoring to other cardiological indications has not been sufficiently explored in scientific trials to date. However, telemonitoring could be beneficial in clinical settings by improving discharge processes, such as accelerating patient discharge following rhythmological interventions and enhancing aftercare. Notably, telemedical supervision may facilitate faster and more reliable uptitration of guideline-directed medical therapy, taking into account patients’ clinical tolerability and vital signs. The STRONG-HF trial highlighted this potential, demonstrating a significantly reduced risk of HF readmission, and alleviated symptoms when guideline-directed medical therapy was uptitrated within 2 weeks of discharge, alongside monitoring of the clinical status and evaluation of laboratory values [53].

In the context of a prehospital setting, the ongoing randomized controlled study, ResKriVer-TAVI, funded by the Federal Ministry for Economic Affairs and Climate Action (German Clinical Trials Register DRKS00027842), is investigating whether telemedical interventional management enhances clinical outcomes, specifically cardiovascular hospitalization and death from any cause, in patients with aortic stenosis awaiting transcatheter aortic valve implantation [54].

While feasibility studies demonstrate the proof of concept for the latest technologies, there is a critical need for randomized trials to evaluate the clinical efficacy of cutting-edge telemonitoring devices and their eventual integration into routine practice. As it stands, it remains unclear whether noninvasive measurements obtained through wearable devices are equally effective compared with implanted cardiac devices. Additionally, the potential advantages of combining methods that integrate multiple sensors have yet to be tested against a single-sensor approach for superiority.

#### AI-Supported Applications in Telemonitoring and Remote Patient Management

As the number of patients eligible for telemedical care continues to grow, machine learning tools hold significant promise for addressing the challenges of numerical scaling and the preprocessing of complex health data from diverse sources. Currently, triaging vital parameters is an established practice. In the future, advancements in pattern recognition are expected to enhance prioritization and improve the prediction of individual decompensation thresholds.

Nevertheless, AI applications are currently not eligible for standard cardiovascular telemedicine care. With the implementation of the European AI Act in March 2024 [55], clear transparency requirements and specific obligations for higher-risk AI applications will create a wide range of opportunities for research into their applications in telemonitoring patient care.

AI is also a promising tool for identifying novel HF parameters: AI applications could integrate smartwatch-based data with established intracardiac and extracardiac device monitoring data. A real-time analysis of wearable-derived intrinsic hemodynamic changes (such as heart rhythm, heart rate, level of activity, pedometer data, and oxygen saturation) could further enhance patient-related outcomes by providing instant alerts to the telemedical center.

The telemedical sensor system will likely be complemented by automated speech analysis to detect pulmonary congestion, as promising data showed a prediction of HF events up to 3 weeks in advance [56]. The “Cordio HearO” smartphone app was introduced in a pilot study including 40 patients with acute cardiac decompensation [57]. This innovative strategy is currently investigated as part of the “Telemed5000-Stimme” observational study (DRKS00020763) in patients on regular hemodialysis [58] in Germany, as well as the “VAMP-HF” study (AI-Based Voice Analysis for Monitoring Patients Hospitalized With Acute Decompensated Heart Failure) in patients with acute decompensated HF at the Deutsches Herzzentrum der Charité and the US Mayo Clinic [59].

Besides their promising transformative potential, the implementation of AI tools in cardiovascular telemonitoring remains a challenge in terms of ethical scenarios and data protection.

## Discussion

The development of telemedicine for the care of patients with HF in Germany has been an extremely long and difficult process, paved by public funding. Important milestones are major pioneering studies such as the TIM-HF2 and IN-TIME trials, which demonstrated the effectiveness of telemedicine approaches using various technological methodologies. The success of these initiatives was largely due to the close cooperation between researchers and technology developers, who created innovative solutions for clinical practice. One significant hurdle that had to be overcome was the traditionally strong separation between inpatient and outpatient care settings in Germany.

With the gradual introduction of telemedical services for patients with HF, research in this area has not come to an end. Instead, the knowledge gained will be used to continuously improve care and advance the digital transformation of the health care system. Care research approaches, such as the forthcoming TIM-HF4 study, play a crucial role in evaluating the efficacy of noninvasive telemonitoring in a real-life setting, particularly regarding health economic criteria.

As a future approach, the establishment of telemedical care for patients with HF across Europe is likely to benefit from the experiences gained in Germany. Despite the lengthy journey, these efforts have resulted in significant improvements in the care of patients with HF.

## Abbreviations

AI: artificial intelligence
BfArM: Bundesinstitut für Arzneimittel und Medizinprodukte
CHF: chronic heart failure
CIED: cardiac implantable electronic device
CRT-D: cardiac resynchronization therapy with a defibrillator
DGIM: Deutsche Gesellschaft für Innere Medizin
DGK: Deutsche Gesellschaft für Kardiologie
DiGA: Digital Health Application
ECG: electrocardiogram
ECG: European Society of Cardiology
G-BA: Federal Joint Committee (Gemeinsamer Bundesausschuss)
HF: heart failure
INH: Interdisciplinary Network for Heart Failure
IN-TIME: Influence of Home Monitoring on Mortality and Morbidity in Heart Failure Patients with Impaired Left Ventricular Function
IQWiG: Institut für Qualität und Wirtschaftlichkeit im Gesundheitswesen
LVEF: left ventricular ejection fraction
mHealth: mobile health
NYHA: New York Heart Association
PAP: pulmonary artery pressure
TIM-HF: Telemedical Interventional Monitoring in Heart Failure Study
TIM-HF2: Telemedical Interventional Management in Heart Failure II
